# Successful Treatment of Refractory Diamond-Blackfan Anemia Using Metoclopramide and Prednisolone

**DOI:** 10.5505/tjh.2012.80008

**Published:** 2012-06-15

**Authors:** Barış Malbora, Zekai Avcı, Namık Özbek

**Affiliations:** 1 Baskent University, School of Medicine, Department of Pediatric Hematology, Ankara, Turkey

## TO THE EDITOR

Diamond-Blackfan anemia (DBA) is a rare congenital hypoplastic anemia characterized by severe normochromic- macrocytic anemia, reticulocytopenia, a decrease in the number or absence of bone marrow erythroid precursor cells, and normal megakaryocytic and granulocytic differentiation [1,2,3,4]. Herein we report the successful treatment of steroid-refractory DBA in a 10-month-old male using prednisolone plus metoclopramide. 

The patient was referred to our hospital for evaluation of severe anemia. The first indication of severe anemia occurred 2 months earlier. He received red blood cell transfusion at that time. Physical examination at admission to our hospital showed the infant was pale. Laboratory findings were as follows: hemoglobin (Hb) level: 4.5 g dL^–1^; mean corpuscular volume (MCV): 73 fL; red cell distribution width: 21.6%; reticulocyte count: 0.44%; white blood cell count: 14.9 x 10^9^ L^–1^; platelet count: 391 x 10^9^ L^–1^. Hypochromic microcytic erythrocytes, normochromic erythrocytes, macrocytic erythrocytes, and normal platelets were also noted. Biochemical and urine analyses were normal. Hb electrophoresis showed an elevated HbA2 level in the patient (4.1%) and in his father (4.3%), which was compatible with the β-thalassemia trait. The patient’s red cell adenosine deaminase level (1321 IU L^–1^) was high (reference: 788 IU L^–1^). Bone marrow aspiration biopsy showed decreased erythropoiesis, with normal myeloid precursors and megakaryocytes. 

**Figure 1:**
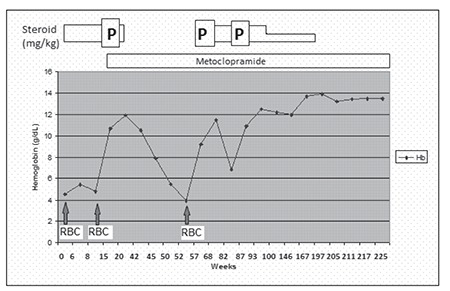
The patient’s hemoglobin values and the drugs he wasadministered. PS: Pulse steroid; RBCT: red blood cell transfusion.

The patient was given prednisolone 2 mg· kg^–1^·d^–1^ for 1 month, but did not respond, and was then treated with a tapered regimen of high-dose methylprednisolone (30 mg· kg^–1^·d^–1^ for 3 d, followed by 20 mg· kg^–1^·d^–1^ for 4 d), and again no improvement was observed. The patient received red blood cell transfusion twice during corticosteroid therapy to treat severe anemia. Due to resistance to steroid treatment, we initiated metoclopramide 0.3 mg·kg^–1^·d^–1^ (3 times daily). On the day 28 of metoclopramide therapy the patient’s Hb level increased to 10.7 g dL^–1^. Steroid treatment was reduced, and then withdrawn after metoclopromide therapy was initiated. The patient’s Hb level was 5.5 g dL^–1^ and his reticulocyte count was 2.4% after 6 months of metoclopromide treatment. Pulse steroid was given, the steroid dose was reduced to 1 mg·kg^–1^·d^–1^, and the metoclopromide dose was increased to 0.5 mg·kg^–1^·d^–1^. Metoclopramide was decreased to 0.2 mg·kg^–1^·d^–1^ (twice daily administration) and steroid was decreased to 0.25 mg·kg^–1^·d^–1^ after 2 years of treatment. At the time this report was prepared the patient had been receiving metoclopramide without steroids for 7 months, and his Hb level was within normal limits (Figure 1). 

Several medications have been used to treat both steroid- dependent and steroid-refractory DBA patients [1,4]. Metoclopramide induces prolactin release, which improves erythropoiesis indirectly—possibly mediated via bone marrow microenvironmental cells released by the pituitary gland [5,6]. Recently, metoclopramide has been shown to be an effective therapy for DBA [5,6,8]. Abkowitz et al. [5] reported that a DBA patient of theirs with pure red cell aplasia transiently improved during pregnancy and lactation. This improvement was associated with an increase in the secretion of prolactin. Following this index case, other researchers used metoclopramide to treat 9 patients with DBA that were refractory to other therapies, and 3 of them responded. Metoclopramide was successfully used in the presented patient, who did not respond to treatment with standard-dose and high-dose methylprednisolone. 

The adverse effects of metoclopramide include agitation, fatigue, headache, extrapyramidal reactions, and hyperprolactinemia. It was reported that hyperprolactinemia has a carcinogenic effect in rodents and humans; it also increases the risk of breast cancer in pre- and post-menopausal women. Nonetheless, the relationship between hyperprolactinemia and prostate cancer is not as strong as its relationship with breast cancer [9]. The relationship between the serum prolactin level and cancer has not been established in childhood, which necessitates further research. Treatment with metoclopramide was effective in the presented patient. Because this agent is relatively inexpensive and safe for patients with DBA that are refractory to steroids, we think that metoclopramide can be considered a drug of choice for patients with DBA. 

**Acknowledgement **

Oral and written informed consent was obtained from the patient’s parent. The present case report protocol conformed to the principles outlined in the Declaration of Helsinki (1975) and later revisions, and was approved by the Baskent University, School of Medicine Ethics Committee, Ankara, Turkey.

**Conflict of Interest Statement **

None of the authors have any conflicts of interest, including specific financial interests, relationships, and/ or affiliations, relevant to the subject matter or materials included in this manuscript.
